# Exploring the Impact of Physical Therapy on Patient Outcomes Across the Cancer Care Continuum: A Narrative Review

**DOI:** 10.7759/cureus.87144

**Published:** 2025-07-01

**Authors:** Maheshkumar Baladaniya, Shraddha Baldania

**Affiliations:** 1 Department of Physical Therapy, Neighborhood Physical Therapy PC, New York, USA; 2 Department of Physical Therapy, Enjoy Rehab PT PC, New York, USA

**Keywords:** cancer care, carcinoma, neoplasm, physical therapy, quality of life

## Abstract

Cancer and its treatments frequently lead to functional impairments, physical limitations, and reduced quality of life (QOL), posing significant challenges for patients across the cancer care continuum. Physical therapy (PT) is a critical intervention to mitigate these effects, from diagnosis through survivorship and palliative care. This narrative review synthesizes evidence on PT’s role in improving functional capacity, managing treatment-related side effects, and enhancing overall well-being in cancer patients and survivors. A comprehensive literature search was conducted on PubMed, ScienceDirect, and Google Scholar using specific keywords and Boolean operators to identify peer-reviewed studies on PT interventions across all cancer care phases. Findings highlight that structured PT interventions, including prehabilitation, exercise programs, and specialized techniques like complete decongestive therapy, significantly reduce pain, fatigue, lymphedema, and deconditioning while improving mobility and QOL. For example, prehabilitation enhanced surgical outcomes in functional capacity, and PT during treatment reduced cancer-related fatigue. The review also underscores PT’s psychosocial benefits, such as reduced anxiety and depression, and its cost-effectiveness in long-term cancer rehabilitation. However, challenges like inconsistent integration, limited access, and lack of standardized referral pathways persist. Multidisciplinary collaboration and early PT integration are essential to optimize outcomes. This review advocates for evidence-based PT strategies to enhance patient outcomes and QOL, emphasizing the need for further research to address implementation barriers and standardize protocols.

## Introduction and background

Cancer is considered a global health challenge, as the data reports 19 million new cases and 10 million deaths in 2020, and the disease currently affects over 50 million people [1,2]. However, globally, socioeconomic, ethnic, and racial differences exist despite various developments in screening, early identification, and intervention of cancer [3,4]. These differences persist at each phase of cancer care partly due to limited involvement and access to healthcare services, insufficient or unequal distribution of resources, restricted access and involvement with medical services, financial constraints, lack of health insurance, and geographic inaccessibility to care [5,6]. These difficulties may start as soon as a patient is diagnosed and persist throughout their course of treatment, aftercare, and survivorship. Advancements in cancer care have led to increasingly complex interventions involving multistep evaluations, multimodal therapies, and the diagnosis of screening anomalies and cancer symptoms [7,8].

In addition to the primary interventions, physical therapy (PT) is a non-pharmacological intervention that is necessarily prescribed for inclusion in the overall cancer care [9]. Exercise is highly advised for people with cancer and has the added benefit of being affordable [10]. According to the United States Centers for Disease Control and Prevention [11], exercise is a subset of physical activity that is designed to maintain or enhance one or more aspects of physical fitness. It is essential in mitigating the side effects of cancer and its treatments by alleviating fatigue and pain, enhancing mobility, improving quality of life (QOL), and facilitating recovery post-treatment. However, the integration of PT into standard oncology care is often inconsistent, with limited accessibility, lack of standardized referral pathways, limited awareness among healthcare providers, and disparities in access to rehabilitation services [12,13]. Hence, this narrative review aims to comprehensively explore the effectiveness of PT on patient outcomes across different stages of cancer care, highlighting its essential contributions and identifying existing gaps in research and clinical implementation.

## Review

**Figure 1:**
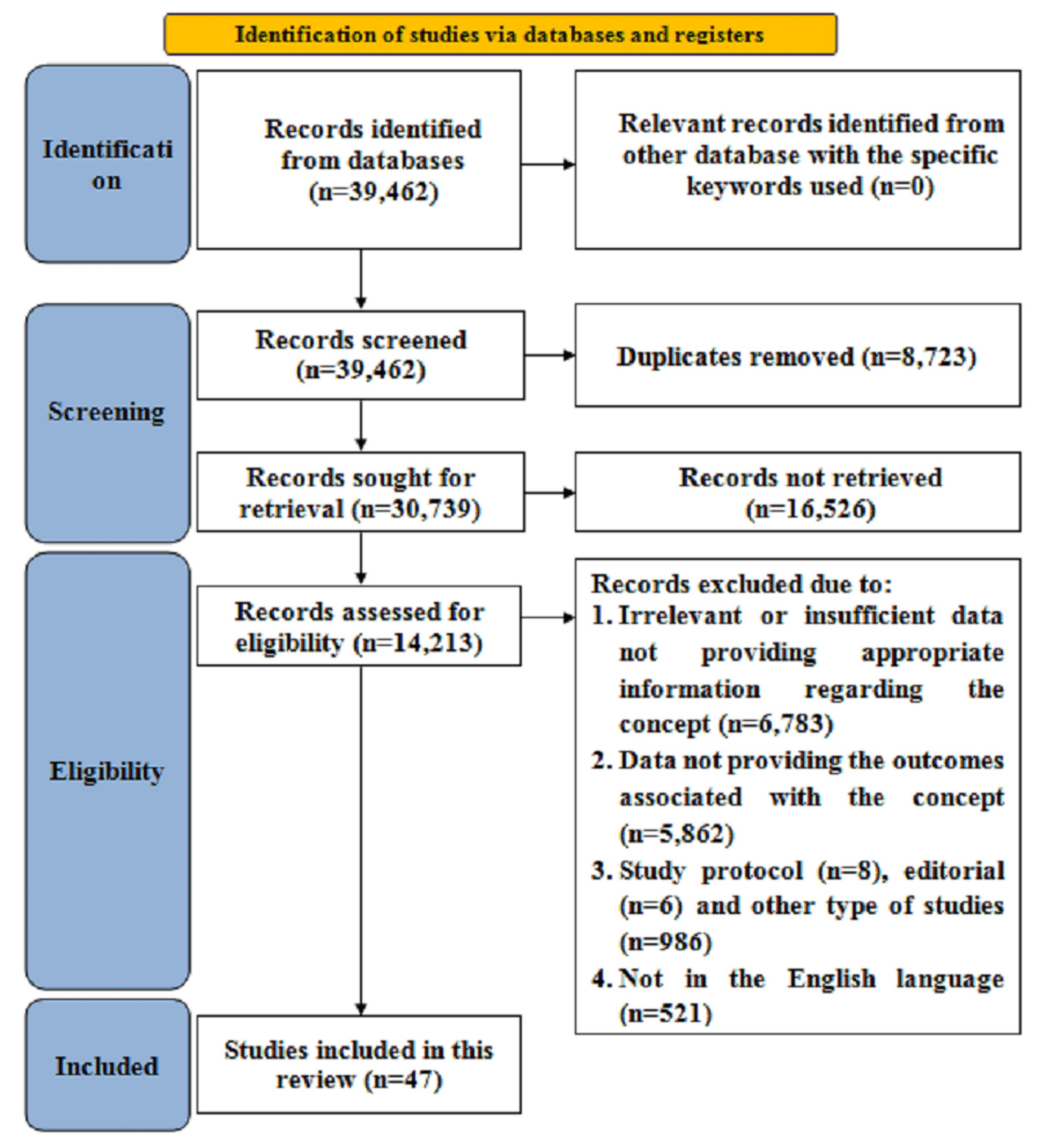
Search strategy

The Scale for the Assessment of Narrative Review Articles (SANRA) guided article selection, inclusion/exclusion criteria, critical appraisal, and evidence presentation [14] along with the Preferred Reporting Items for Systematic Reviews and Meta-analyses (PRISMA) framework [15], as illustrated in Figure 1.

Data sources and search strategy

A thorough literature search was conducted on PubMed, ScienceDirect, and Google Scholar databases from inception to February 2025, incorporating keywords with Boolean operators that consisted of, “Physical therapy”, OR “Physiotherapy”, AND “Cancer care”, OR “Carcinoma”, OR “Neoplasams”, AND “Patient outcome assessment” AND “Treatment outcome evaluation”.

Study screening and selection

The inclusion criteria involved studies that concentrated on evaluating the role of PT interventions at any phase of the cancer care continuum, including PT in pre-treatment phase, during cancer treatment, and in post-treatment cancer survivorship, research articles assessing functional outcomes, QOL, pain management, mobility, or psychological benefits associated with PT in cancer care, peer-reviewed articles, editorials, conceptual articles, and published reports, open access studies published in English. However, investigations that focused solely on pharmacological or surgical interventions without reference to PT, studies lacking an available English translation or not published in English, with only a title and no abstract, those without open access, and those lacking sufficient knowledge with respect to the context were excluded.

**Figure 2:**
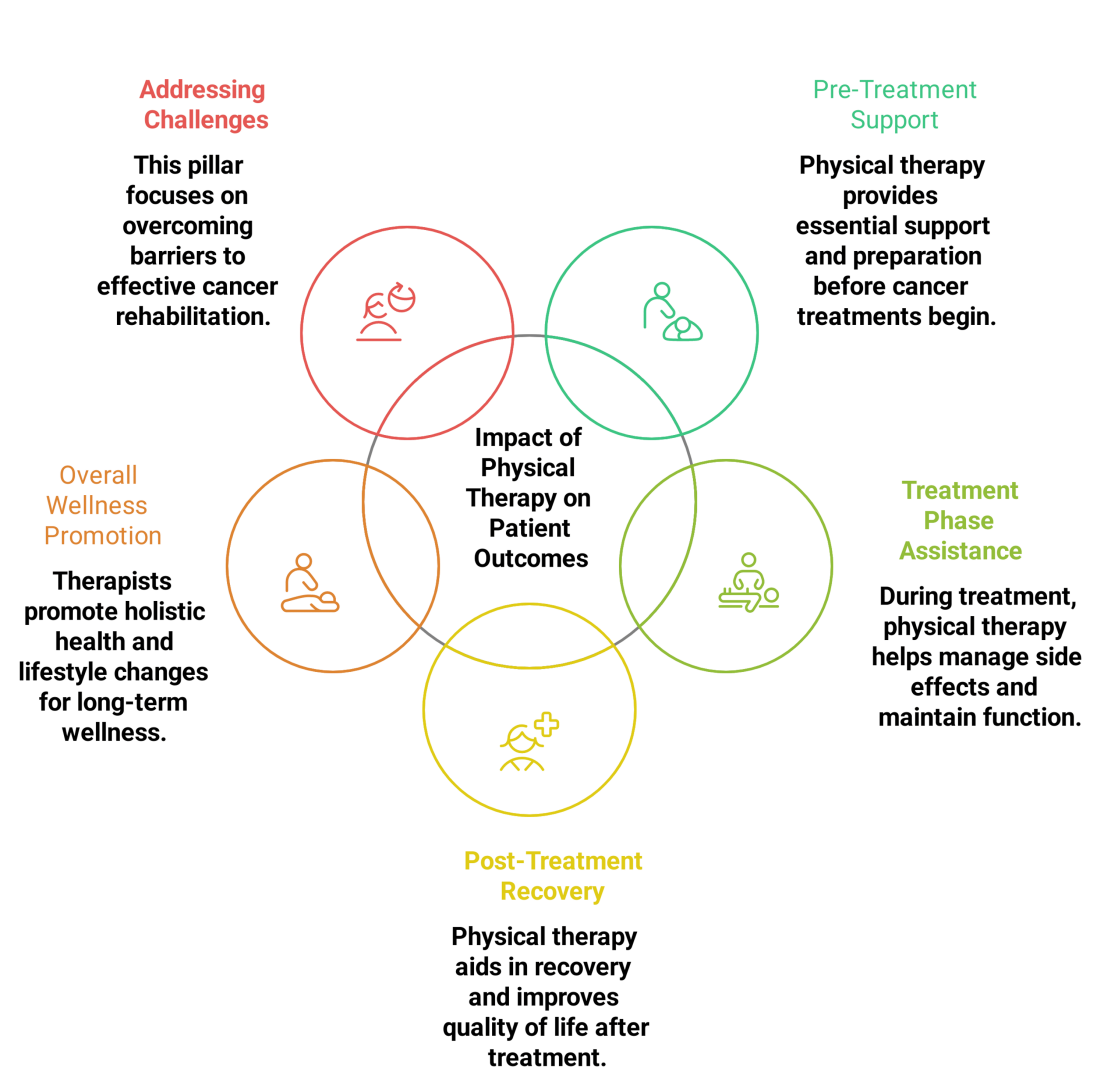
Overview of impact of physical therapy on patient outcomes The figure is created by the authors illustrating the impact of physical therapy on patient outcomes across the cancer care continuum.

Two reviewers independently assessed article eligibility for inclusion. To remove duplicates, titles and abstracts were screened initially, followed by a second review to exclude ineligible studies and a full-text assessment to confirm inclusion. Reviewer disagreements were resolved through discussion and consensus. To synthesize the findings, a critical narrative approach was employed [14]. The studies involved diverse methodologies and outcome measures, leading to substantial heterogeneity. Figure 2 illustrates an overview of the impact of PT on patient outcomes.

Data extraction and synthesis

The data was extracted based on the information that concentrated on evaluating the role of PT interventions at any phase of the cancer care continuum, including PT in pre-treatment phase, during cancer treatment, and in post-treatment cancer survivorship, and research articles assessing functional outcomes, QOL, pain management, mobility, or psychological benefits associated with PT in cancer care. The critical narrative technique was utilized to incorporate figures and text to summarize and validate evidence.

Physical therapy in the cancer diagnosis and pre-treatment phase

Cancer rehabilitation, involving a multidisciplinary team with physical therapy (PT) and occupational therapy as core components, aims to enhance cognition, gait, activity participation, and functional status while reducing morbidity and improving quality of life (QOL) [16,17]. In the diagnosis and pre-treatment phase, PT addresses physical and psychosocial impairments through baseline assessments of function, mobility, and strength, informing personalized exercise programs and patient education to maintain function during treatment [16,17]. Prehabilitation PT optimizes physical capacity, improves surgical outcomes, reduces complications, and accelerates recovery. For example, Rucińska et al. (2022) found that multimodal prehabilitation, including exercise, nutrition, psychological support, and smoking cessation, reduced hospital stays, complications, stress, and depression [18]. Similarly, Silver et al. (2013) reported a 20% improvement in functional capacity (measured by the 6-minute walk test) in breast cancer patients undergoing pre-surgical aerobic and resistance training [19]. PT also mitigates anxiety, depression, and fatigue through tailored exercises and manual therapy, fostering psychological resilience [16,17]. However, access to prehabilitation is limited by inconsistent protocols and reimbursement barriers, necessitating standardized guidelines and cost-effectiveness studies [20]. Thus, PT is integral to comprehensive cancer care, preparing patients for treatment by enhancing physical and emotional well-being [20,21].

Physical therapy during cancer treatment

Annually, 3.5 million suffer from daily pain, and 4.5 million cancer patients die, yet few receive proper pain management [22]. Pain affects 30-50% of cancer patients, with 75-90% experiencing severe pain that impacts their QOL [23]. Advancements in diagnosis and treatment have extended the lives of cancer patients; however, pain significantly reduces their QOL [24]. Physical therapists offer non-pharmacological therapies to reduce pain in palliative care [24]. Physical therapists serve as a vital component of palliative care, improving QOL and function while managing symptoms like pain, limited range of motion, weakness, balance, coughing, and dyspnoea [25,26].

PT during cancer treatment restores function, manages side effects, boosts energy, enhances well-being, and supports long-term survivorship. By designing individualized exercise programs, physical therapists help patients improve range of motion, regain strength, and enhance balance, ultimately aiding in the restoration of independence in daily activities. Additionally, PT techniques involving modalities (heat or cold therapy), manual therapy, and therapeutic exercises effectively mitigate side effects like lymphedema, pain, neuropathy, and joint stiffness [27]. A meta-analysis by Pérez et al. (2023) of 15 trials (n=1,200) found that PT interventions, including aquatic therapy and resistance training, reduced cancer-related fatigue by 30% (effect size=0.65, p<0.001) and improved mobility by 18% in patients undergoing chemotherapy [27]. Lymphedema management, a common issue in breast cancer, benefits from PT techniques like complete decongestive therapy (CDT), which reduced arm volume by 35% in a cohort study of 80 patients [28]. Despite these benefits, PT integration during treatment is inconsistent due to limited oncologist referrals and patient fatigue, highlighting the need for automated referral pathways and patient education to enhance uptake [29].

Engaging in physical therapist-guided physical activity can increase energy levels, counteract fatigue, and improve overall strength and endurance. Moreover, PT provides emotional support, releases endorphins to improve mood, reduces anxiety and depression, and enhances the overall emotional well-being of patients. By concentrating on flexibility exercises, strength training, and cardiovascular conditioning, PT supports both short-term recovery and long-term survivorship by preserving function, preventing deconditioning, and reducing future health risks [26].

Physical therapy in post-treatment cancer survivorship

Physical therapy (PT) is pivotal in post-treatment cancer survivorship, aiding recovery after surgery, radiation, and chemotherapy by restoring physical function, improving mobility, and enhancing quality of life (QOL) [30,31]. Personalized exercise programs help survivors regain strength, flexibility, and balance, supporting independence in daily activities and addressing survivorship-specific challenges, such as return to work and late-onset effects [30]. A systematic review by Sleight et al. (2022) (n=25 studies, 2,500 participants) found that combined aerobic and strength training improved QOL by 22% (p=0.002) in breast, prostate, and colorectal cancer survivors [30]. Mano et al. (2022) reported enhanced physical function and endurance in childhood cancer survivors through long-term rehabilitation [32]. Novel interventions, like yoga-based PT, reduced fatigue and improved sleep quality in breast cancer survivors [33]. PT also mitigates late-onset effects, such as osteoporosis, with weight-bearing exercises increasing bone mineral density by 5% in pelvic cancer survivors [34]. Survivorship PT facilitates return to work by improving functional capacity and managing chronic pain or neuropathy, yet barriers like limited access in rural areas and lack of survivor-specific guidelines persist, necessitating telehealth and policy advocacy [35,36].

Tailored care plans, integrating exercise, nutrition, and patient education, address late effects like bone pain, fractures, or vaginal stenosis post-pelvic radiation, reducing long-term complications [37,38]. Beyond physical benefits, PT significantly impacts psychosocial and cognitive outcomes in cancer patients. Exercise programs, including aerobic and mind-body interventions like tai chi, reduce anxiety and depression by promoting endorphin release [36]. PT also addresses chemotherapy-related cognitive impairment (“chemo brain”), with studies showing improved attention and memory; for instance, Campbell et al. (2021) reported a 12% improvement in cognitive function in 70 breast cancer patients after a 12-week exercise program [39]. Psychosocial benefits extend to reducing fear of recurrence, with group-based PT fostering social support. Barriers include stigma around mental health and a lack of integrated psychosocial services in oncology. Research is needed to optimize PT’s role in addressing cognitive and emotional challenges across the cancer continuum [36,39].

Physical therapy in palliative and end-of-life care

Physical therapy plays a vital role in palliative and end-of-life care, focusing on symptom management, comfort, and QOL for patients with advanced cancer. Interventions include gentle exercises (e.g., stretching, low-intensity mobility training), positioning techniques to reduce pressure ulcers, and breathing exercises to alleviate dyspnea. A systematic review by Lowe et al. of 12 studies (n=800) found that PT in palliative care reduced pain by 25% (p=0.04) and improved QOL scores by 18% in patients with metastatic cancer [36]. PT also supports caregivers by teaching safe transfer techniques, reducing physical strain, and improving the confidence of participants in hospice settings [39]. Challenges include limited therapist training in palliative care and restricted access in hospice settings, particularly in low-resource areas. Future research should focus on standardizing palliative PT protocols and integrating them into hospice care models [39].

Telehealth and technology in cancer rehabilitation

Telehealth and technology are transforming cancer rehabilitation by improving access to PT, especially for patients in rural or underserved areas. Telehealth platforms enable remote PT consultations, delivering tailored exercise programs via video calls. Telehealth-based PT improved physical function in cancer survivors compared to usual care [40]. Wearable devices, such as fitness trackers, monitor activity levels and enhance adherence; a study by Galiano-Castillo et al. (2022) found a 20% increase in exercise compliance among 100 breast cancer survivors using wearables [41]. Emerging technologies like virtual reality (VR) show promise for pain management; a pilot study reported a 30% reduction in pain perception during VR-based PT sessions [42]. However, barriers like digital literacy, internet access, and reimbursement for telehealth services limit adoption. Future efforts should focus on equitable access and integrating technology into standard PT protocols.

Promoting overall wellness and a healthy lifestyle

By incorporating healthy lifestyle choices such as staying active, maintaining a balanced diet, managing stress, getting enough rest, and practicing self-care, individuals can improve and enhance their treatment journey. A balanced diet rich in lean proteins, vegetables, fruits, and whole grains can improve energy levels, strengthen the immune system, and aid in recovery [43].

Moreover, regular physical activity, tailored to individual abilities and treatment stages, can improve mood, help manage side effects, and boost overall health. Effective stress management techniques like deep breathing, meditation, and relaxation exercises can help patients navigate the emotional challenges of cancer. Adequate rest is essential for healing and overall well-being, contributing to improved mood, immune function, and QOL. By embracing self-care practices and making healthy lifestyle choices, cancer patients and survivors can take an active role in their health, potentially improving treatment outcomes and long-term health [43].

Multidisciplinary collaboration and referral pathways

Cancer care necessitates collaboration among medical oncology, surgery, radiation therapy, and support services for optimal patient outcomes [44]. The Institute of Medicine advocates for team-based, patient-centered models, emphasizing quality reporting, a well-coordinated workforce, and evidence-based practices [45]. Effective cancer care demands seamless communication, information sharing, and coordination among patients, specialists, primary care physicians, and support services across multiple treatments and providers [20]. Physical therapists are essential in restoring function, managing treatment side effects, and enhancing the QOL for cancer patients and survivors. The early involvement of physical therapists bridges care gaps, supports shared decision-making, and empowers patients, enhancing treatment adherence [31].

Integrating PT into oncology teams enhances patient care and outcomes across the cancer continuum. The Clinically Integrated Physical Therapist (CI-PT) model embeds physical therapists in oncology clinics to proactively preserve mobility during cancer treatment. This model includes routine mobility screening, personalized evaluations, and targeted PT interventions [20]. Integrating physical therapists into oncology teams enables personalized care, early detection of functional changes, and targeted interventions. The CI-PT model standardizes rehabilitation with a patient-centered, data-driven approach, ensuring appropriate care [20,31]. Hence, successful integration requires collaboration among physical therapists, rehabilitation administrators, and oncology teams for seamless care. The CI-PT model enhances patient care, QOL, and long-term outcomes for cancer survivors [20].

Despite its benefits, PT in cancer care remains underutilized, emphasizing the need for improved referrals and provider awareness [31]. Hence, optimizing referrals based on patient and provider feedback ensures timely, personalized PT for cancer survivors. Moreover, multidisciplinary collaboration, education, and integration of physical therapists into clinical teams improve referrals and enhance cancer rehabilitation [46]. Promoting exercise counseling, provider education, and targeted referral strategies enhances rehabilitation access, reduces barriers, and improves cancer survivor outcomes [47].

Effective cancer care relies on interdisciplinary communication and coordinated support for patients. Collaboration among healthcare providers, including social workers, nurses, and oncologists, is vital to meeting cancer patients' and survivors' diverse needs. Integrating social work and nurse coordination into oncology teams enables proactive, patient-centered care and tailored support across the cancer continuum [35]. Multidisciplinary team meetings, like those in the Victorian framework, provide a structured platform for care coordination and addressing cancer patients' physical and psychosocial needs. Strengthening information sharing, multidisciplinary teams, and care coordination ensures holistic, patient-centered cancer care, optimizing treatment and outcomes while reducing service duplication [26,48].

Challenges, barriers, and future directions

Post-treatment survivorship care varies based on location, professional culture, and available resources [49]. Late diagnosis and drug resistance are significant challenges in cancer care [50]. Additionally, El-Deiry WS et al. reported that disparities arise due to socioeconomic factors, limited access to care, and a lack of diversity in genomic databases, which impacts cancer research. To address these issues, community outreach programs tailored to diverse racial and ethnic groups, along with lifestyle modifications and vaccination initiatives, are essential for cancer prevention [51] Furthermore, patient barriers to clinical trial participation have been recognized, with navigators assisting through referrals, transportation arrangements, and proactive patient support [52].

With longer survival, cancer patients require post-treatment care [52]. Many cancer survivors experience long-term physical and psychological challenges, including fatigue, pain, neuropathy, anxiety, depression, relapse fear, work difficulties, financial strain, and reduced QOL [53,54]. Cancer care faces numerous challenges, including rising diagnoses impacting patients, providers, and healthcare systems, with over 2 million new cases expected in 2024 [54]. Barriers to effective cancer care include role recognition, multidisciplinary coordination, care transitions, specialist-primary care communication, unequal access, and resource limitations. Overcoming these barriers requires patient-centered interventions informed by experiences and perspectives on cancer care coordination [55]. 

Availability and accessibility of cancer rehabilitation services

The sources offer key insights into the accessibility and availability of cancer rehabilitation services, highlighting both advancements and challenges in this critical aspect of cancer care. Efforts to standardize cancer rehabilitation and integrate it into oncology care have been significant, with organizations like the American College of Surgeons Commission and the National Comprehensive Cancer Network (NCCN) emphasizing the importance of comprehensive rehabilitation services for cancer survivors [56].

Moreover, telehealth has emerged as a promising avenue to improve accessibility to cancer rehabilitation services, particularly for patients facing challenges related to in-person care, such as those in rural areas or with poor internet connectivity. While telehealth offers a means for patients to receive essential rehabilitation services, concerns remain regarding accessibility and the importance of in-person care, underscoring the need for a balanced approach that considers individual preferences and circumstances [57].

Overall, the field of cancer rehabilitation has seen significant growth and recognition, with a focus on optimizing the QOL, maximizing function, and addressing the diverse physical, mobility, and cognitive problems that can arise during and after cancer treatment. By addressing barriers to access, leveraging telehealth where appropriate, and ensuring a comprehensive approach to rehabilitation services, healthcare systems can enhance the availability and accessibility of cancer rehabilitation services, consequently enhancing the quality of care and outcomes for cancer survivors [57].

Education of patients, caregivers, and healthcare providers

Patients and caregivers can have a major impact in educating healthcare providers and sharing their perspectives, experiences, and expectations to improve the quality of care. Patients and caregivers believe that participating in healthcare education enhances collaboration and strengthens patient-provider relationships, provides direct feedback, and influences curricula development for healthcare professionals [58]. Initiatives that involve patients early in the education process of healthcare providers can lead to better understanding, improved communication, and a more patient-focused model of care [58].

Furthermore, patient education is essential in enabling people to comprehend their diseases, available treatments, and methods of self-care. Effective health education empowers patients to understand medical information, make informed decisions, and engage in their care, improving outcomes and reducing readmissions [58]. By providing understandable and actionable information, healthcare providers can encourage patient-led health management, partner with care providers, and navigate their healthcare journey more effectively. Some examples of such initiatives include the Cancer Care Ontario approach for selecting, implementing, and evaluating patient-reported outcome measures for routine clinical use in cancer. It also assists caregivers to co-develop educational resources and training for providers, fostering patient-centered care across the cancer continuum [59]. Similarly, the Cancer.Net platform by the American Society of Clinical Oncology offers oncologist-approved resources, including videos and podcasts, to educate patients and caregivers on cancer management, enhancing informed decision-making [60]. Moreover, programs like the Alberta Cancer Exercise (ACE) initiative provide community-based exercise education for cancer survivors, improving physical function and quality of life through tailored, evidence-based training [61]. 

In summary, the education of patients, caregivers, and healthcare providers is a collaborative process that fosters mutual understanding, enhances communication, and promotes patient-centred care. By involving patients and caregivers in healthcare provider education, ensuring accessible and actionable patient education materials, and promoting active patient engagement, healthcare systems can improve care quality, patient outcomes, and overall healthcare experiences for all stakeholders involved. The need to address the gap in oncology rehabilitation care is underscored, with a significant percentage of cancer patients requiring physical rehabilitation but only a small proportion accessing these services [62]. Bridging this gap and increasing access to rehabilitation services is critical for fulfilling the rehabilitation requirements of cancer patients and survivors.

Advocacy and policy implications

Coverage for rehabilitation services differs significantly among payers, yet these services remain underutilized, with many eligible patients not receiving referrals. Enhancing service implementation and addressing barriers like limited patient awareness and insufficient physician engagement are essential [62]. To address these challenges, several steps are necessary, including implementing standardized exercise oncology protocols to define eligibility and outcomes, clarifying provider roles across the care continuum, aligning advocacy efforts with policy opportunities, integrating implementation strategies from the outset, and developing a research framework to evaluate the effectiveness and cost-efficiency of rehabilitation services [63].

The consensus recommendations highlight the necessity of developing and evaluating personalized rehabilitation interventions, along with brief assessment tools to identify individual needs in cancer rehabilitation [64]. Additionally, they emphasize the importance of educating clinicians, patients, and families on the significance and effectiveness of cancer rehabilitation [64]. CancerCare advocates for state and federal policies that guarantee equitable access to high-quality, safe, and affordable cancer treatment for all Americans. It also works to prevent discriminatory insurance practices and supports shared decision-making in cancer treatment planning, ensuring patient values and priorities are respected [60,61]. In summary, advocacy and policy efforts for cancer rehabilitation should focus on ensuring access to affordable exercise and rehabilitation services, improving service implementation, developing and evaluating personalized rehabilitation interventions, and educating clinicians, patients, and families on the significance and effectiveness of cancer rehabilitation.

## Conclusions

This narrative review has comprehensively explored and synthesized the existing evidence supporting PT's invaluable contributions to optimizing patient outcomes across all phases of the cancer care continuum. From the pre-treatment phase through active treatment, survivorship, and palliative care, physical therapists play an integral role in addressing impairments, managing side effects, promoting functional recovery and QOL, and providing rehabilitative care for individuals with cancer. Evidence shows that PT interventions, including prehabilitation, exercise, education, and specialized treatments, enhance surgical readiness, reduce treatment side effects, improve well-being, restore daily function, and promote self-management. Moreover, physical therapists play a vital role in palliative rehabilitation, enhancing comfort, dignity, and QOL for patients nearing the end of life. Despite its proven benefits, cancer rehabilitation remains underutilized due to limited access, weak referral pathways, and insufficient awareness among patients, caregivers, and healthcare providers. Successful education initiatives can address these gaps. For example, Cancer Care Ontario’s approach to selecting, implementing, and evaluating patient-reported outcome measures for routine clinical use in cancer also engages caregivers to co-develop educational resources and training, fostering patient-centered care. Similarly, Cancer.Net by the American Society of Clinical Oncology offers oncologist-approved resources, including videos and podcasts, to educate patients and caregivers on cancer management, enhancing informed decision-making. The Alberta Cancer Exercise (ACE) initiative provides community-based exercise education for cancer survivors, improving physical function and QOL through tailored, evidence-based training. Research gaps persist, including limited data on long-term PT outcomes, optimal intervention timing, and cost-effectiveness across diverse cancer populations. Future studies should investigate standardized PT protocols, telehealth integration, and culturally tailored interventions to enhance access and equity. As cancer care continues evolving towards more multidisciplinary, longitudinal models, there is a pressing need for PT to become seamlessly embedded across the full continuum of oncology care pathways. Integrating PT from diagnosis to survivorship or end of life enhances patient rehabilitation, improving function and QOL. This review highlights its essential role in comprehensive cancer care, emphasizing the need for continued implementation, advocacy, and research.
